# Ethnic Identity Protects and Internalized Racism Harms Health and Coping in Asian Americans Following COVID-19 Discrimination: A Mixed-Methods Study

**DOI:** 10.1007/s40615-024-02000-5

**Published:** 2024-04-22

**Authors:** Emily R. Nhan, Aisha R. Williamson-Raun, Rachel Chan, Joyce P. Yang

**Affiliations:** 1 Department of Psychology, University of San Francisco, San Francisco, CA USA; 2 Institute for Clinical and Economic Review, Boston, MA USA; 3 National Alliance On Mental Illness, San Francisco, CA USA

**Keywords:** Ethnic identity, Internalized racism, Discrimination, COVID-19, Racial trauma, PTSD

## Abstract

Mechanisms underlying the link between COVID-19 anti-Asian racial discrimination and psychological health are underexplored. This mixed-methods study examined the moderating effects of ethnic identity and internalized racism on the relationship between COVID discrimination and behavioral health outcomes among Asian Americans. We hypothesized that individuals with lower ethnic identity and higher internalized racism levels would demonstrate more adverse outcomes, including worsened psychological trauma and identity-avoidant behaviors, post-discrimination. Asian American participants (*N* = 215) responded to a Qualtrics survey, including qualitative and quantitative questions on COVID-related racism experiences, ethnic identity, internalized racism, trauma, and other subsequent effects. For qualitative analysis, participants were sorted into four subgroups defined by low- and/or high-ethnic identity and internalized racism scores, and we explored themes in participant reports of identity-related coping effects after racism. We additionally used hierarchical multiple regression analyses to quantitatively assess the moderating impact of ethnic identity and internalized racism on the relationship between COVID discrimination and trauma. Analyses revealed no moderating effects from the two identity variables. However, qualitative analyses identified themes of identity-promoting and identity-avoidant behavioral responses, and moderation analyses revealed that ethnic identity had a main effect on mitigating racial trauma, while internalized racism exacerbated both racial trauma and PTSD levels. This study identified ethnic identity and internalized racism as underlying causes to behavioral health outcomes for Asian Americans. Results offer mental health providers serving Asian clients insight into identity-related influences to help optimize culturally appropriate interventions and support initiatives of identity promotion to foster community engagement for this population.

## Introduction

The sudden surge of the COVID-19 pandemic in December 2019 was followed shortly by a similar spike in stigma and racism against Asian targets and communities worldwide [1, 2]. In the U.S., hate crimes against Asians increased by over 300% from 2020 to 2021, and nearly half of Asian adults in the U.S. have reported experiencing at least one racist incident since the onset of COVID [3, 4]. These hate crimes have occurred in numerous settings and have varied in severity, including incidents such as online harassment, vandalism of Asian businesses and property, and physical assaults and murders in public areas. Asian elderly, women, and families have been targets of assault and homicide [5–10]. Additionally, former President Trump and other political representatives have publicly used misinformative language and labeled COVID-19 the “Wuhan virus” and “Kung flu,” further contributing to the increased xenophobia and the negative sentiment towards Asians.

Extensive literature has studied the psychological outcomes of discrimination, but more recent research has captured these impacts specifically in the context of anti-Asian racism during the pandemic. Studies show that anti-Asian hate leads to increased mental health challenges [11–14], as Asian individuals who have gone through a discriminatory experience are 30% more likely to exhibit distress [15]. These studies have identified a variety of psychopathological and behavioral health effects, including anxiety, depression, and substance use disorders [2, 11, 12, 16]. In the short-term period following an instance of discrimination, those who experienced COVID-19-related racial discrimination were at an increased risk of binge drinking, non-suicidal self-injury, and suicidal ideation [17]. Additionally, targets experiencing discrimination report increased posttraumatic stress disorder (PTSD) and racial trauma symptoms, further exhibiting distress, hypervigilance, and isolation from others, as well as fear and worry for their safety [14]. A scoping review of 35 studies evaluating the mental and physical effects of COVID-19 discrimination found that all reported negative impact on health outcomes, including worsening subjective well-being, internalizing and externalizing symptoms, sleep, and physical health in addition to the effects described above [18].

Given the deleterious mental effects of racism on Asian American individuals, it is crucial to understand the mechanisms through which racial discrimination impacts psychological well-being and behaviors. Existing literature has described a host of variables that impact the relationship between experiencing discrimination and psychological sequelae in populations with minoritized identities. For example, Asian American individuals with high rejection sensitivity have been found to experience increased stress when faced with racial microaggressions [19]. Negative family environments and residing in ethnically homogenous communities are also risk factors for negative mental impacts post-discrimination, exacerbating feelings such as anxiety, loneliness, and isolation [20]. Concurrently, qualities such as acculturation, positive self-thought, higher education, family cohesion, and strong social networks serve as protective factors for Asian Americans, reducing the occurrence and intensity of psychological disorders [1, 21, 22].

Ethnic identity, described as the level of affiliation and connectedness with one’s ethnic group, has emerged in literature as a protective factor buffering psychological sequelae of racist experiences. For example, ethnic identity pride was found to be a moderator between discrimination and improved mental health, such as lower depression levels [23]. Research suggests that ethnic identity affirmation, pride, attachment, and sense of belonging all have positive associations with well-being, including experiences of acceptance and positive relationships with others for validation [24]. Through a strong understanding of one’s ethnic background, with its history and accomplishments, individuals have an improved sense of belonging with their minority in-group, which may decrease the need to belong with the majority outgroup; as a result, they are equipped with improved psychological resources to better cope with and manage racially discriminatory experiences [25–27]. For the Asian American population, greater affiliation with one’s ethnic identity is related to other adaptive outcomes such as improved school performance, self-esteem, and health-promoting behaviors [12, 28]. A strong sense of ethnic identity has demonstrated benefit for ethnic and racial socialization processes, resulting in posttraumatic growth and healthy coping practices to protect against mental challenges [20, 21, 29].

Conversely, internalized racism is commonly observed as a risk factor in the relationship between discrimination and psychological sequelae. Internalized racism is a process in which racially oppressed groups believe the negative race-related views set upon them by the dominant group and, as a result, reinforce racial oppression. When individuals adopt racist messages and ideas of their own racial group, they are exposed to internalizing these beliefs into their self-concept, consequently leading to negative mental health consequences [30]. When internalized racism and identity beliefs merge, individuals can experience further distress in the form of anxiety and depression [31]. Similarly, there are increased risks of negative self-esteem and psychological and physiological stress responses when experiencing devaluation of one’s racial group and developing internalized racism [32, 33]. Among Asian Americans in particular, internalized racism was related to negative psychosocial consequences such as general distress, anxiety, and depression, as well as poorer self-esteem and quality of life [12, 34–36]. However, the moderating impact of internalized racism is not consistent across subgroups in this population. One study found that internalized racism effects after COVID racism varied by generational status, exacerbating somatic symptoms in first-generation Asian Americans, while mitigating anxiety and depression symptoms for 1.5 and second-generation Asian Americans [37]. Among Asian American women who frequently experienced discrimination, greater levels of internalized racism were associated with lower stress levels, suggesting the need to further investigate this construct in this population [38].

It is important to explore the intersection of the two identity-related constructs, ethnic identity and internalized racism, due to their opposing effects on self-concept. Past research has highlighted how Asian Americans may experience a complex navigation of their identity in the U.S., managing “a fractured reality” of success expectations perpetuated by the model minority myth, while also confronting a “fractured identity” from the difficulty adjusting to conflicting social norms, obligations, and expectations [39]. Additionally, literature has discussed implications that internal racism and acceptance of oppression can result from racism, while acknowledging that ethnicity, gender identity, and socioeconomic status are notable factors intersecting with racist experiences and resulting psychological challenges [40]. Due to oppressive experiences, identity is questioned frequently and can cause individuals to believe they are inferior, doubt themselves, and exhibit self-hatred. Previous literature suggests that during the process of identity-conflicting experiences, in attempts to create a sense of belonging with the oppressors, individuals tend to create responsive strategies to distance themselves from their racial group, including internalized racism, disidentification, and “othering” [30]. Often facing a unique yet difficult position amidst racial differences in America (i.e., Black and White), Asian Americans’ racist experiences commonly arise from stereotypes and unrealistic expectations of their behaviors in society [12, 41, 42]. As a result, they are subjected to social rejection and harassment when they do not conform to such social expectations [12, 43–46].

Although there is existing literature on experiences of racism and psychological sequelae of Asian Americans, this community is still absent from many dialogues and existing research, which has focused primarily on Black and White racial experiences. Additionally, there is emerging, yet insufficient, research regarding mechanisms (e.g., ethnic identity and internalized racism) underlying mental health for the Asian American community. Lastly, scant literature has examined these factors affecting Asian psychological well-being within the context of COVID-19 racism; therefore, our study adds to critical understanding of Asian Americans experiences of racism and the short- and long-term impacts.

As the first mixed-methods study to explore concurrent risk and protective factors affecting mental health after COVID-19 anti-Asian discrimination, we sought to thoroughly and accurately assess participants’ experiences and outcomes. We utilized a combination of qualitative and quantitative data collection and analyses, allowing for data triangulation and a better understanding of the phenomena [47]. We first collected qualitative data, which allows participants to thoroughly describe the emerging phenomenon’s complexity in their own words. In doing so, their personal narratives emphasize how participants process and make meaning of their experiences. Simultaneously, we collected data using psychometrically validated measures to quantitatively assess how participant reports of racial trauma and PTSD symptoms were affected by ethnic identity and internalized racism.

This study explored the impact of two identity factors, ethnic identity and internalized racism, on the relationship between COVID-19 anti-Asian discrimination and subsequent mental health behaviors. We hypothesized that Asian individuals with low levels of ethnic identity and high levels of internalized racism would display more adverse mental health challenges in the form of higher racial trauma and PTSD scores, as well as more ethnic identity-avoidant behaviors.

## Methods

### Participants and Data Collection

Our study included adult participants over the age of 18 who self-identified as Asian and were living in the U.S. for the majority for 2020. From September to December 2020, *n* = 70 college students from a West Coast university were enrolled in the first wave of the study through an online SONA system for course credit. In January 2021, an additional *n* = 145 participants were recruited in a second wave convenience sample through online flyers distributed within Asian interest groups, which consisted mostly of young Asian professionals; subjects were given $15 Amazon gift cards for study completion. The university ethics review board reviewed and approved all study procedures, which involved completing an online consent form, followed by a 1–1.5 h online Qualtrics survey of qualitative open-ended free response questions as well as quantitative questionnaires.

### Qualitative Measures

Participants were first asked to respond with “yes” or “no” to the question, “1) After the coronavirus pandemic, which started in December of 2019, did you experience discrimination due to your race/ethnicity?” If they responded “yes,” they were then asked the following questions: “2) Please describe your experiences”; “3) Please describe how these experiences impacted you in the short and long term”; and “4) How, if at all, have the nature of these experiences changed as the pandemic has evolved?”.

### Quantitative Measures

#### Ethnic Identity

The Revised Multigroup Ethnic Identity Measure (MEIM) [48] is a 12-item measure of the degree to which a person identifies with their own ethnic group. This scale contained the following three factors: (1) affirmation/belonging, (2) ethnic identity achievement, and (3) ethnic identity behaviors. The MEIM is scored on a 4-point Likert scale ranging from 4 (Strongly agree) to 1 (Strongly disagree). Scores were averaged to obtain the global MEIM score. The measure has shown high internal consistency and validity in previous studies [48–50] and demonstrated very good internal consistency in this study (*α* = 0.88).

#### Internalized Racism

The Internalized Racism Scale For Asian Americans (IRSAA) [51] is a 152-item measure of the degree to which one agrees with stereotypical beliefs about their Asian American ethnic group. The scale contained the following five factors: (1) sense of inferiority, (2) endorsement of negative Asian American stereotypes, (3) desire to be “more White,” (4) desire to distance oneself from those who fit the negative stereotypes (within-group discrimination), and (5) minimization or denial of racism. Twenty-one items from this scale were selected to assess internalized racism more relevant to the context of COVID-related racism. The IRSAA is scored on a 6-point Likert scale ranging from 1 (Strongly disagree) to 6 (Strongly agree). Scores were averaged to obtain the global IRSAA score. The scale has shown high reliability and validity in previous research [51] and showed acceptable internal consistency in this study (*α* = 0.77).

#### Racial Trauma

The Trauma Symptoms of Discrimination Scale (TSDS) [52] is a 21-item measure of the frequency of trauma symptoms following an experience of discrimination, including the following factors: (1) uncontrollable distress and hyperarousal, (2) alienation from others, (3) worry about safety and the future, and (4) being keyed up and on guard. The TSDS is scored on a 4-point Likert scale ranging from 1 (Never) to 4 (Often). Scores were summed to obtain the global TSDS score and subscales. This measure has shown excellent reliability in previous research [52] and demonstrated excellent internal consistency (*α* = 0.96) in this study.

#### PTSD

The Posttraumatic Stress Disorder Checklist for the DSM-5 (PCL-5) [53] is a 20-item measure of the presence and severity of PTSD symptoms a person may experience in the past month. The PCL-5 is scored on a 5-point Likert scale ranging from 0 (Not at all) to 4 (Extremely). Scores were summed to obtain the global PCL-5 score. The scale has shown excellent reliability and validity in previous research [53] and showed excellent internal consistency (*α* = 0.97) in this study.

### Qualitative Analysis

We used conventional qualitative analysis [54] to explore the extent to which participants discussed ethnic identity and internalized racism constructs in their narratives of experienced COVID-related discrimination and resulting psychological and behavioral effects. Our team engaged in “checks and balances” during the analytic process by engaging in reflexivity and discussing biases, as suggested by consensual qualitative research theory [55]. The research team consisted of six members, five of whom identified as women and one who identified as a man. Coding was led by a researcher working for a nonprofit research institute, while four team members were undergraduate research assistants; coding procedures were supervised by an assistant professor of psychology at a West Coast university. Team members identified as African and Asian American, with Chinese, Filipinx, Indian, Malaysian, Taiwanese, and Vietnamese backgrounds.

After preparation, the five team members created a preliminary codebook that aggregated, through discussion, the meaningful codes observed by each member. Coders paired up to read through each transcript together, meeting with the fifth supervisory member to discuss and resolve discrepancies, as is standard in qualitative content analysis [56].

When final coding was completed, the team combined codes that were related in content and context to generate categories and explore underlying themes that connected categories together. To assess differences in behavioral health outcomes in participants with different levels of ethnic identity and internalized racism, using a median split for both measures [57], participants were sorted into one of four subgroups for comparison: low ethnic identity/low internalized racism (LELI), low ethnic identity/high internalized racism (LEHI), high ethnic identity/low internalized racism (HELI), and high ethnic identity/high internalized racism (HEHI). We then separated these participant responses into four separate transcripts and evaluated differences in the codes between each of the transcripts. Significant differences in mental health and identity-related descriptions between the more “extreme” LEHI and HELI groups would indicate the presence of the two factors (i.e., ethnic identity and internalized racism) as moderators. For this component of the analysis, participants who did not respond to both ethnic identity and internalized racism measures were excluded.

### Quantitative Analyses

Hierarchical multiple regression analyses were conducted to assess the combined moderating influences of ethnic identity and internalized racism on the relationship between anti-Asian racism and the following trauma outcomes of (1) racial trauma and (2) PTSD. In preliminary steps, we performed assumption checks of normality, and dummy-coded the predictor variable assessing whether participants experienced COVID racism, to assign numerical value to the responses of no (0) or yes (1), and labeled this variable “racism.” At the first step, the predictor of racism and the identity variables, ethnic identity and internalized racism, were entered. Next, to assess moderation effects, interaction terms were entered at the next four steps: Step 2) racism × ethnic identity; Step 3) racism × internalized racism; Step 4) ethnic identity × internalized racism; and Step 5) racism × ethnic identity × internalized racism. If there is a statistically significant change in *R*^2^ at a step where an interaction term was entered, then the variables in that interaction term are considered effective moderators.

## Results

### Descriptive Statistics

Participants (*N* = 215) were identified as Asian American/Asian (90.7%) or mixed-race Asian (9.3%) (Table 1). Fifteen ethnicities were represented in the sample, with the most frequent endorsements being Chinese (33.0%), multi-ethnic Asian (13.5%), Filipinx (12.1%), Korean (9.3%), Taiwanese (7.9%), and Vietnamese (7.4%; see Table 1). Participants’ gender identification included female (68.3%), male (27.9%), and genderqueer, non-binary, or another self-reported identification (3.8%). Majority of the sample (89%) were US nationals, while the remaining participants were international students or non-US nationals living in America. Participants ranged in US geographical location, with a small majority living on the West Coast (56.9%), 15.3% on the East Coast, 12.9% in the Midwest, 6.2% in the Southern States, and 4.3% in Alaska, Hawaii, and other non-mainland territories. *N* = 178 participants completed the entire Qualtrics survey, while *n* = 60 participants responded “yes” and *n* = 118 participants responded “no” to experiencing discrimination.

**Table 1:** Demographics of study sample [12]

Demographic characteristic	*N*	*%*^a^	*M*	*SD*
Age	215		24.99	10.41
Gender	208			
Female		68.3		
Male		27.9		
Non-binary, genderqueer, other		3.8		
Monoracial/multi-racial	215			
Asian		90.7		
Mixed-Asian		9.3		
Nationality	209			
U.S		89.5		
Non-U.S		10.5		
International student	209			
Yes		8.6		
No		91.4		
Educational level	207			
Not in college		0.5		
First year		34.3		
Second year		9.7		
Third year		7.2		
Fourth year		5.8		
Fifth year or graduate student		30.9		
Graduate or professional school		11.1		
Other		0.5		
Geographic location (in past 6 months)	209			
US West Coast		56.9		
US Midwest		12.9		
US Southern States		6.2		
US East Coast		15.3		
US non-mainland (e.g., islands, Hawaii, Alaska)		4.3		
Outside U.S		4.3		
Self-described ethnicity	215			
Asian American		3.7		
Cantonese		0.5		
Chinese		33		
Filipino/a		12.1		
Hmong		0.9		
Indian		2.3		
Japanese		4.7		
Korean		9.3		
Punjabi		0.5		
Singaporean		0.5		
Sri Lankan		0.5		
Taiwanese		7.9		
Vietnamese		7.4		
Multiethnic		13.5		
Other		3.3		

^a^% = adjusted percent of sample who endorsed the answer

Asian participants who experienced racism had a mean ethnic identity (MEIM) score of 1.70 (*SD* = 0.47), while those who did not experience racism had a mean score of 1.91 (*SD* = 0.43) (Table 2). These ethnic identity means are notably lower than MEIM levels observed in Asian subgroups of other studies, such as Brown et al. 2014 (*M* = 3.65, *SD* = 0.71) and Phinney 1992 (*M* = 3.02, *SD* = 0.45) [58, 59]. Conversely, those who experienced racism had minimally higher internalized racism (IRSAA) scores (*M* = 2.56, *SD* = 0.71) compared to those who did not experience racism (*M* = 2.47, *SD* = 0.54). These means were slightly lower than that observed in previous literature (*M* = 2.91, *SD* = 0.56) [51]. Asian participants who responded “yes” compared to participants who responded “no” to experiencing COVID racism had significantly higher racial trauma (TSDS) (*M* = 50.6, *SD* = 14.9 vs. *M* = 38.3, *SD* = 12.6) and PTSD (PCL-5) scores (*M* = 29.8, *SD* = 22.6 vs. *M* = 17.2, *SD* = 14.6) (*p* < 0.001 for all scales) [14].

**Table 2:** Descriptive statistics of identity measures and trauma outcomes

	Participants who experienced discrimination (*n* = 60)		Participants who did not (*n* = 118)	
Measures	*M*	*SD*	*M*	*SD*
Multigroup Ethnic Identity Measure	1.70	0.47	1.91	0.43
Internalized Racism Scale For Asian Americans	2.56	0.71	2.47	0.54
Trauma Symptoms of Discrimination Scale (TSDS)*	50.6	14.9	38.3	12.6
Posttraumatic Stress Disorder Scale (PCL-5)*	29.8	22.6	17.2	14.6

*Scale is scored by summing all item scores (instead of taking an average)

The means, standard deviations, and intercorrelations of the independent and dependent variables are presented in Table 3.

**Table 3:** Means, standard deviations, and intercorrelations among identity and trauma variables

Variable	1	2	3	4
1. Ethnic identity (MEIM)	-	-	-	-
2. Internalized racism (IRSAA)	0.21**	-	-	-
3. Racial trauma (TSDS)	− 0.23**	0.15*	-	-
4. PTSD (PCL)	− 0.07	0.17*	0.52***	-
*M*^a^	1.83	2.50	42.5	21.4
*SD*	0.46	0.60	14.6	18.6

^a^*N* = 178. *p < 0.05, **p < 0.01, ***p < 0.001

### Qualitative Results

Using a median split on ethnic identity and internalized racism scores, participants were separated into one of the following four subgroups: LELI, LEHI, HELI, and HEHI (*n* = 9 to 21). We observed no differences in reported psychological effects or identity-related coping behaviors between the four subgroups, suggesting no apparent moderating effects of ethnic identity and internalized racism on subsequent mental health behaviors. However, we still aimed to examine how the constructs of ethnic identity and internalized racism generally influenced the participants’ behavioral outcomes, specifically those that indicated either adaptive or adverse coping responses following a racist incident. Qualitative coding and analyses derived several behavioral effects emerging from ethnic identity and internalized racism that demonstrated either distancing or increasing in proximity to one’s ethnic or racial identity.

#### Adverse Outcomes

##### Guilt About Ethnicity and Self-Blame

 Participants reported a multiplicity of adverse cognitive outcomes as a result of discriminatory experiences, including feelings of self-blame and responsibility for the COVID-19 pandemic. Some described feeling like “my ethnicity was forced to hold responsibility for something we didn’t mean to start” (18/19-year-old Filipino-Chinese American man; Modesto, CA). Others felt that “There were so many people saying that somehow East Asian people deserved this and honestly a part of me agreed with them because our communities are somewhat racist” (18/19-year-old Taiwanese American non-binary person; Chicago, IL), and some even reported that the Asian community “seems like they are not only easy targets but also justifiable targets of discrimination to the racist and malicious” (early 20 s Chinese American man; San Francisco and Los Angeles, CA). Other participants reported a similar phenomenon of feeling guilt and shame for being Asian or being associated with the Asian community, saying, “I felt guilty for being categorized as Asian because many people began to fear and blame Asians for starting the COVID-19 pandemic” (18/19-year-old Filipina American woman; South San Francisco and Fairfield, CA), and another expressed “I think on more of a subconscious level it’s made me more self-conscious/ashamed of my identity, but I’d never admit that to people outside of my racial/ethnic in-group” (early 20 s Chinese American woman; San Francisco and Los Angeles, CA).

##### Feeling Targeted, Self-Conscious, and Hypervigilant

 The constant incidents of discrimination led numerous participants to feel like they were being alienated and othered by perpetrators and, as a result, became increasingly hyper-aware of themselves in public settings. Participants described feeling like an outsider, discussing that they “felt very targeted” (mid-40 s Korean American woman; Phoenix, AZ), as well as “unsafe, targeted, and unwelcome in social groups…Very careful and uneasy taking public transit” (18/19-year-old Chinese American woman; Alameda and San Francisco, CA). One participant expressed that “it was upsetting because I do my job to help people, but now I was seen as a walking public health threat” (early 20 s Japanese-Brazilian American woman; San Francisco, CA), while another felt “like a terrorist in my own hometown” (18/19-year-old Filipinx American non-binary person; San Francisco, CA).

Participants expressed feeling increasingly aware of both their behaviors and appearances in public, especially health-oriented behaviors. Participants consistently reported feeling “self-conscious with my body language, making sure not to touch my face or even cough in the slightest” (18/19-year-old Filipinx American non-binary person; San Francisco, CA) and “afraid that if I sneezed or coughed that it would trigger someone again, especially because I was Asian” (late 20 s Korean Filipina woman; Seattle, WA). An early 20 s Filipina American woman residing in San Francisco, CA, and Honolulu, HI, described, “Whenever I got too close to someone or coughed even just a little bit, I noticed the people around me giving me the stink eye or looking at me very weird.” Other participants were hyperconscious of other health-related behaviors such as masking, expressing that they “didnt feel safe wearing a mask or afraid ppl would be rude to me bc they think i look chinese” (late 20 s Vietnamese American woman; Santa Ana, CA).

Participants began engaging in additional protective behaviors following a discriminatory experience, being more cautious and guarded in their day-to-day routines. Many participants modified their grocery shopping routines, with one participant reporting, “Now we don’t go inside the stores to shop” (late 30 s Japanese American woman; Ames, IA), and another participant stating, “My family and I switched entirely to grocery delivery so that we wouldn’t have to grocery shop in public” (mid-20 s Chinese American non-binary person; New Orleans, LA). Other participants altered their modes of transportation, detailing that “ever since that incident, I’ve tried to walk in busy areas and drive more” (late 20 s Korean-Filipina American woman; Seattle, WA). Others have modified other daily activities as a result of their racist experience, including one participant who reported “I stopped walking my dog alone in my own neighborhood” (early 30 s Taiwanese American woman; Seattle, WA).

##### Losing Individuality

 Participants observed that they were being grouped together regardless of their ethnicity and felt that they were losing their individuality and unique identities. Examples include, “Them discriminating against me made me feel grouped in and like I was losing my own individuality” (18/19-year-old Filipina-Chicana American woman; Napa and Vacaville, CA), and “The racism and xenophobia also became unruly and terrifying because they didn’t care who you were or what ethnicity you were” (18/19-year-old Filipina American woman; Sacramento, CA). A couple of participants further explained “I felt very targeted. I felt that people didn’t care about the nuances that I was from a different country but instead, lumped all asians into their hatred” (mid-40 s Korean American woman; Phoenix, AZ), and “Although the virus began in China, I noticed that people began to see all Asians as one ethnicity, rather than individualizing each one” (18/19-year-old Filipina American woman; South San Francisco and Fairfield, CA).

##### Downplaying of Experience or “Playing” Along with Racism

 Some participants responded to situations of discrimination by downplaying or justifying their experiences, in part normalizing these incidents. For example, a late 20 s Korean-Filipina American woman living in Seattle, WA, articulated, “In general, I try not to attribute people’s actions to racism or discrimination unless they outright say a racist slur.” Another respondent reported, “I try to keep my head below all of it and brush any negative emotions to the side, almost as if I was forgiving the person” (18/19-year-old Japanese American woman; Los Angeles and San Francisco, CA).

Other participants claimed they were not affected by these experiences of discrimination. An early 20 s Filipina American woman who lived in San Francisco and San Diego, CA, stated, “I did not care much for it because I assumed that I experienced these things due to the coronavirus. I always justified experiences like this with the virus.” Similarly, others expressed, “I’ve learned to just not take it personally and ignore it” (mid-20 s Chinese American man; Minnesota) and “Long term, it has not really impacted me…I am not surprised and I just carry on” (early 20 s Korean American woman; Birmingham, AL and Pittsburgh, PA).

There were also reports of participants “playing” or going along with racism through actions of tolerating, condoning, or cooperating with the racism. Some participants, in response to racist jokes, laughed it off, detailing that “honestly, in the moment, I thought it was funny” (early 20 s Taiwanese American woman; Carmel and Bloomington, IN) and “my friends make jokes about it, but none of them are actually being racist” (18/19-year-old Chinese man; Kansas). Other examples include, “A guy across the street yelled ‘coronavirus!’ I mocked him back by looking around and saying, ‘Who? Me?’ and doing a little jig” (late 20 s Korean-Filipina American woman; Seattle, WA).

##### Reduced Salience of Asian Identity

One major behavioral change frequently observed among Asian targets resulting from their experience was the conscious effort to engage in less Asian-oriented behaviors and conceal their Asian appearance, distancing themselves from their Asian identity or community. For instance, one participant said “I usually stop speaking Japanese and switch to English if possible” (18/19-year-old Japanese American woman; Syracuse, NY, and Koriyama and Fukushima, Japan). Another reported that “when I go out in public, I try to minimize my appearance (wearing a hat, mask, sunglasses) so that it’s not as easy to identify that I am Asian” (mid-40 s Korean American woman; Phoenix, AZ).

##### Distrust of American and Its Institutions

Participants shared that after experiencing COVID discrimination, they began to lose trust in American institutions, stating, “I feel resentful towards racist government officials and the racial environment” (18/19-year-old Chinese American woman; St. Louis, MO, and Lexington, KY). Some participants expressed an increased distrust in not only American systems but in America as a country, saying, “It was a reminder of what I had long known about America’s pattern in scapegoating a group of non-White people during times of crisis” (mid-30 s Chinese-Thai American woman; Arlington, VA) and “This just left me a bad impression of this country as a whole” (early 20 s Chinese-Indonesian American woman; San Ramon and Oakland, CA).

#### Adaptive Outcomes

##### Collective Identity and Further Connection with Culture

 Conversely, following discriminatory incidents, many participants developed adaptive coping responses that helped promote connection with their ethnic identity and, as a result, improve their overall well-being. Some participants reported feeling a stronger connection with their cultural community. One stated “I don’t know if this fully counts as my experience because it was towards the Chinese community in general, but I associate myself apart of that community” (18/19-year-old Chinese American woman; Elk Grove, CA). Often, when some members of their ethnic group experienced discrimination, participants empathized with the targets of the incidents, expressing “I felt annoyed and disgusted, not just for me but for other asian Americans who have to go through experiences like this as well” (early 20 s Filipina American woman; San Francisco, CA, and Honolulu, HI). Another participant even shared sympathy for affected Asian businesses, expressing “I am more saddened by the multiple Asian companies and restaurants that were attacked with hoaxes and rumours which ultimately ruined their businesses” (18/19-year-old Chinese Indonesian woman; San Francisco and Los Angeles, CA and Jakarta, ID). One participant attempted to further explore and connect with their Asian culture, saying they “became more interested in speaking Chinese and watching Chinese television” (early 30 s Taiwanese American woman; Seattle, WA).

##### Empowerment and Defending of Identity and Culture

Participants reported feeling stronger, more confident, and more in control of their lives as a result of discrimination. For instance, one said “Now I have me guard up. I will not allow anyone to attack me just because of my skin color” (late 40 s Asian American woman; Philadelphia, PA and Atlantic City, NJ). Another participant felt more compelled to stand up in defense for their ethnic and cultural group, explaining that “Seeing other coronavirus-related racism online really angered me, and I felt emboldened to stand up to shitheads if the time ever came” (late 20 s Korean-Filipina American woman; Seattle, WA).

When racist perpetrators blamed Asian people for the cause of COVID-19, many participants denounced their views, defending that “people were ignorant and didn’t do their research at all and just took it out on my community” (18/19-year-old Chinese American woman; Elk Grove, CA) and that “Some Americans wanted someone to blame for this pandemic instead of collaborating together like a nation should be” (18/19-year-old Filipino-Chinese American man; Modesto, CA). Participants pointed out how “non-asian countries in Europe/North America have more cases of covid and are less able to control the spread” (early 30 s Taiwanese American woman; Seattle, WA) and “Since most Asian countries have been handling the virus so well, I think other ethnicities have started to eat their words a little bit” (18/19-year-old Japanese American woman; Honolulu, HI). Others have noted the hypocrisy in the treatment of other groups who have further spread COVID, saying “after the European outburst, no one asks if a white person has recently been to Europe and deny access if he/she has been to Europe” (early 30 s Chinese woman; San Francisco, CA).

##### Increased Comfort and Presence in Asian/Communities of Color Areas

Many participants expressed feeling more trustful in Asian communities or spaces with more people of color and less comfortable outside of their in-group. For instance, one stated “I think in the long term it has made me more distrustful of people outside my ethnic group” (early 20 s Chinese-Mexican American man; Naperville, IL, and St. Louis, MO) and “specifically, I’ve been much more wary of white people” (early 20 s Chinese American woman; San Francisco and Los Angeles, CA). Another participant adjusted their daily habits to avoid conflict, reporting “I feel that I have avoided many of these experiences since I only shop at an Asian market nowadays since I would feel uncomfortable when I would go to Walmart or Target or Trader Joe’s” (early 20 s Filipina American woman; San Francisco and San Diego, CA).

##### Community-Oriented Behaviors

Following their discriminatory experiences, participants also expressed how they began to seek comfort and aid from their peers or close friends for social support. One stated that after discriminatory instances, “I remember telling a friend, letting someone know and letting it out. I just carried on with my day, but I do recall letting it out to friends or people willing to listen” (early 20 s Korean American woman; Birmingham, AL, and Pittsburgh, PA).

To combat false narratives of Asian people and people of color, some participants informed or educated others on racist issues. For example, a participant explained “my 17yo niece had to witness and experience this, had a discussion with her about the ignorance of some people and how to educate herself regarding differences and how to be informed on her reactions” (mid-30 s Vietnamese American woman; Garden Grove, Brea, and Laguna Hills, CA), and another stated “I was also more prone to correct people if they said negative things about Asian people” (early 30 s Taiwanese American woman; Seattle, WA). Other participants informed about their racist experiences through other support channels, including a late 20 s Korean-Filipina American woman living in Seattle, WA, who “posted about the incident on social media.”

Some participants became motivated to participate in social justice initiatives, such as “spending time to create a POC startup (with two other Asian women), while taking a leadership role in my Korean American organizing work in the Bay” (mid-30 s Korean American man; San Francisco, CA).

### Quantitative Results

#### Preliminary Analyses

Prior to conducting the moderation analyses, we performed assumption checks of normality, linearity, and homoscedasticity. All dependent variables were measured on a continuous scale, and we had one independent variable and two moderator variables. Through the Durbin-Watson test, we found that the data had no autocorrelation. We also tested for and confirmed a linear relationship between the independent variable and dependent variables. We checked for and observed homoscedasticity through observing the standardized residual plots. The issue of multicollinearity was addressed in multiple ways, including “centering” the moderator variables and checking the variance inflation factor (VIF). There were additionally no outliers or skew, and data and residual errors were normally distributed. Overall, all assumptions for conducting multiple regression analysis were met, and we observed no unusual occurrences in the data. Moreover, we dummy-coded the predictor variable of racism to assign numerical values of 1 or 0 to the yes/no responses.

#### Racial Trauma

First, we conducted a hierarchical multiple regression analysis to determine whether ethnic identity and internalized racism together moderated the impact of COVID racism on racial trauma in participants (Table 4). At the fifth step of the analysis assessing main interaction effects, it was found that ethnic identity and internalized racism did not jointly serve as moderators affecting racial trauma (∆*R*^2^ = 0.002, *p* = 0.515). The same insignificance was observed when assessing individual moderating effects of ethnic identity and internalized racism at the previous Steps 2 and 3 (∆*R*^2^ = 0.0001, *p* = 0.858; ∆*R*^2^ = 0.006, *p* = 0.265, respectively). However, referring back to the first step of the regression assessing main effects, ethnic identity and internalized racism had significant main effects on racial trauma in the predicted directions, with ethnic identity lowering racial trauma scores (*p* = 0.01) and internalized racism exacerbating racial trauma symptoms (*p* = 0.02). While there was an absence of moderators, the two factors still individually influenced racial trauma levels.

**Table 4:** Hierarchical multiple regression moderation analysis of the interactions of racism, ethnic identity, and internalized racism on racial trauma (TSDS)

	Racial trauma (TSDS)^a^					
	*B*	SE *B*	*β*	*R*^2^	∆*R*^2^	*p*-value
Step 1				0.207	-	-
Racism (Yes/No)	10.73	2.14	0.74	-	-	< 0.001
Ethnic identity	− 5.92	2.27	− 0.19	-	-	0.010
Internalized racism	4.12	1.69	0.17	-	-	0.016
Step 2				0.208	0.000	-
Racism (Yes/No)	10.77	2.16	0.74	-	-	< 0.001
Ethnic identity	− 6.23	2.84	− 0.19	-	-	0.030
Internalized racism	4.12	1.70	0.17	-	-	0.016
Racism × ethnic identity	0.83	4.59	0.03	-	-	0.858
Step 3				0.213	0.006	-
Racism (Yes/No)	10.67	2.16	0.73	-	-	< 0.001
Ethnic identity	− 5.67	2.88	− 0.18	-	-	0.051
Internalized Racism	2.32	2.34	0.10	-	-	0.323
Racism × ethnic identity	− 0.32	4.70	− 0.01	-	-	0.946
Racism × internalized racism	3.80	3.40	0.16	-	-	0.265
Step 4				0.216	0.002	-
Racism (Yes/No)	10.68	2.17	0.73	-	-	< 0.001
Ethnic identity	− 5.63	2.89	− 0.18	-	-	0.053
Internalized racism	2.74	2.41	0.11	-	-	0.258
Racism × ethnic identity	− 0.23	4.71	− 0.01	-	-	0.962
Racism × internalized racism	3.36	3.45	0.14	-	-	0.332
Ethnic identity × internalized racism	− 2.40	3.30	− 0.04	-	-	0.467
Step 5				0.218	0.002	-
Racism (Yes/No)	10.41	2.21	0.71	-	-	< 0.001
Ethnic identity	− 5.60	2.89	− 0.18	-	-	0.055
Internalized racism	3.17	2.50	0.13	-	-	0.207
Racism × ethnic identity	− 0.36	4.72	− 0.01	-	-	0.939
Racism × internalized racism	2.94	3.52	0.12	-	-	0.405
Ethnic Identity × internalized racism	− 4.86	5.01	− 0.09	-	-	0.333
Racism × ethnic identity × internalized racism	4.35	6.66	0.08	-	-	0.515

^a^*N* = 178

#### PTSD

We also used a hierarchical multiple regression analysis to assess whether ethnic identity and internalized racism had moderating influences on PTSD in participants following COVID discrimination (Table 5). By Step 5 of the analysis assessing our main interaction term, similarly to the racial trauma outcomes, ethnic identity and internalized racism did not conjointly moderate the relationship between experienced anti-Asian discrimination and subsequent PTSD symptoms (∆*R*^2^ = 0.001, *p* = 0.608), and, at earlier Steps 2 and 3, did not individually moderate PTSD levels (∆*R*^2^ = 0.0.003, *p* = 0.418; ∆*R*^2^ = 0.000, *p* = 0.990, respectively). However, likewise in the racial trauma outcome, both factors’ individual effects on PTSD were in the predicted directions. At the first step of this analysis looking at the main effects of these two factors, internalized racism alone was found to be a predictor of worsened PTSD symptoms (*p* < 0.05), while ethnic identity was not a significant influence (*p* = 0.67).

**Table 5:** Hierarchical multiple regression moderation analysis of the interactions of racism, ethnic identity, and internalized racism on PTSD (PCL)

	PTSD (PCL)^a^					
	*B*	SE *B*	*β*	*R*^2^	∆*R*^2^	*p*-value
Step 1				0.126	-	-
Racism (Yes/No)	11.89	2.87	0.64	-	-	< 0.001
Ethnic identity	− 1.28	3.04	− 0.03	-	-	0.674
Internalized racism	4.89	2.27	0.16	-	-	0.033
Step 2				0.129	0.003	-
Racism (Yes/No)	12.17	2.89	0.66	-	-	< 0.001
Ethnic identity	− 3.13	3.80	− 0.08	-	-	0.411
Internalized racism	4.90	2.27	0.16	-	-	0.032
Racism × ethnic identity	4.98	6.14	0.12	-	-	0.418
Step 3				0.129	0.000	-
Racism (Yes/No)	12.17	2.91	0.66	-	-	< 0.001
Ethnic identity	− 3.12	3.87	− 0.08	-	-	0.421
Internalized racism	4.87	3.14	0.16	-	-	0.122
Racism × ethnic identity	4.97	6.31	0.12	-	-	0.432
Racism × internalized racism	0.05	4.56	0.00	-	-	0.990
Step 4				0.129	0.000	-
Racism (Yes/No)	12.17	2.91	0.66	-	-	< 0.001
Ethnic identity	− 3.12	3.88	− 0.08	-	-	0.424
Internalized racism	4.97	3.24	0.16	-	-	0.128
Racism × ethnic identity	4.99	6.33	0.12	-	-	0.432
Racism × internalized racism	− 0.04	4.64	− 0.00	-	-	0.993
Ethnic Identity × internalized racism	− 0.52	4.43	− 0.01	-	-	0.908
Step 5				0.131	0.001	-
Racism (Yes/No)	12.46	2.97	0.67	-	-	< 0.001
Ethnic identity	− 3.15	3.89	− 0.08	-	-	0.419
Internalized racism	4.51	3.37	0.15	-	-	0.183
Racism × ethnic identity	5.13	6.35	0.13	-	-	0.420
Racism × internalized racism	0.41	4.73	0.01	-	-	0.931
Ethnic identity × internalized racism	2.09	6.74	0.03	-	-	0.757
Racism × ethnic identity × internalized racism	− 4.61	8.96	− 0.07	-	-	0.608

^a^*N* = 178

## Discussion

COVID-19 spurred a rapid increase of anti-Asian racism and has highlighted the need to address these resulting mental health outcomes facing the Asian community. Many recent studies in this area [11, 13, 14] have sought to better understand the relationship between racial discrimination and psychological well-being, but few have accounted for underlying factors that influence this relationship. While existing literature has identified some moderators that impact this relationship, such as in Kim’s (2022) study exploring ethnic identity and critical action, we wanted to explore factors of identity and self-concept (i.e., ethnic identity, internalized racism) and how these contrasting factors conjointly impact subsequent mental health behaviors [12]. This mixed-methods paper adds to emerging research on Asian American mental health, not only by assessing anti-Asian racism and behavioral health outcomes but also by examining two identity-related factors that potentially moderate this relationship in the context of the COVID-19 pandemic.

In our current study, we predicted that participants with lower levels of ethnic identity and higher levels of internalized racism would experience exacerbated PTSD and racial trauma levels and exhibit more identity-avoidant responses and less identity-promoting ones; we predicted the inverse for participants with higher levels of ethnic identity and lower levels of internalized racism. However, findings from both the qualitative and quantitative analyses indicated that ethnic identity and internalized racism were not, individually nor conjointly, moderators influencing the link between COVID-19 anti-Asian racism and behavioral health outcomes.

In our qualitative findings, we observed no differences in the psychological outcomes and identity-related coping behaviors among the four different subgroups, split based on ethnic identity, and internalized racism scores. However, among the overall participant responses emerged several themes, including changes in general mental health and in coping behaviors related to identity. Descriptions depicted a range of reactions, varying from adverse behavioral responses, such as feelings of guilt for being associated with their ethnic group and concealing their Asian identity, to adaptive responses, including defending their culture, seeking social support, and taking part in activism. The spectrum of ethnic identity-oriented responses following a racist incident highlights the potential influences that identity factors, such as ethnic identity and internalized racism, have on the coping and mental health behaviors of the Asian American community. The exact mechanisms of these two identity factors need to be further explored by additional research.

In hierarchical regression analyses for the two trauma-related outcomes, racial trauma and PTSD, all assumptions for conducting multiple regression analyses were run and met, and no unusual occurrences in the data were observed. The final step of the moderation model indicated that neither ethnic identity nor internalized racism was significant moderators affecting the relationship between racism and trauma. However, when observing earlier steps of the regression, Step 1 results demonstrated that ethnic identity and internalized racism individually affected trauma symptoms, with ethnic identity buffering racial trauma scores and internalized racism exacerbating both racial trauma and PTSD levels. This observance of ethnic identity reducing only racial trauma levels and not PTSD may be due to racial trauma better capturing effects of racism, and therefore being more directly racial identity-related than PTSD; strong affiliation with one’s ethnic identity may further protect one’s mental health and promote improved coping responses after facing discrimination [60].

Despite finding that ethnic identity and internalized racism were not moderators, qualitative and quantitative analyses demonstrated that, regardless of exposure to racism, these factors still influenced mental health responses. More specifically, ethnic identity had positive effects on mental health, in the form of adaptive, identity-promoting behaviors and lowered racial trauma levels, and internalized racism worsened health outcomes, inducing adverse, identity-avoidant behaviors and increased PTSD and racial trauma symptoms. Our findings were consistent with existing research, illuminating the harmful psychological effects of anti-Asian racism (e.g., feeling self-conscious or guilty about ethnicity). Furthermore, these results support other previous literature, demonstrating that ethnic identity may serve as a protective factor, likely due to the notion that stronger ties to one’s ethnic group leads to more positive well-being [61], while internalized racism, which involves internalizing negative beliefs about one’s racial group, aggravates mental health. There is not sufficient research exploring the combined moderating effects of ethnic identity and internalized racism together within the context of COVID-19 racism; therefore, our research adds to critical knowledge of Asian Americans experiences of racism and its psychological consequences.

The prevalence of infectious disease-related stigma and discrimination worldwide points to widespread public health concerns that go beyond disease symptoms [62]. It is imperative to leverage the strengths of groups affected by this discrimination, to promote resilience against potential health problems. The identification of ethnic identity and internalized racism as influences on behavioral health outcomes could potentially explain the underlying reason behind some Asian targets’ general mental health challenges. These findings can be useful in helping clinicians who serve Asian American clients better understand how to address the mental health needs of this community. On a broader scale, given that our qualitative results demonstrated community involvement as an adaptive response to discrimination, the current study offers implications for the implementation of identity-promoting interventions at the community level. Specifically, initiatives to encourage and foster identity, in spaces such as public education, research, ethnic minority-oriented spaces, and within families, may increase community knowledge, empower individuals to connect more with their racial in-groups and other ethnic minority groups, and seek to improve community well-being in the form of social support, critical consciousness, and collective action.

### Limitations and Future Directions

#### General Limitations

Our study utilized a convenience sample, consisting of college students enrolled in General Psychology and young Asian-identified participants online, majority of whom received bachelor’s or advanced degrees. Due to this sample, the range of ages in this study skewed young and were relatively educated, potentially affecting generalizability related to age and socioeconomic status. This was also a cross-sectional study, limiting the ability to make causal quantitative inferences about the sequential impacts of racism, ethnic identity, and internalized racism on psychological and identity-related behaviors. Longitudinal or experimental studies in this area could further strengthen the findings of this study. Additionally, this study relied on retrospective self-report measures and qualitative responses to assess outcomes, increasing potential participant bias.

#### Qualitative Limitations

In the subjective coding of qualitative responses and qualitative research methods from five researchers, there are common limitations surrounding neutrality, objectivity, and researcher bias [63]; hence, we utilized a supervisory member to facilitate a larger discussion and validate the coding themes. The splitting of the participants into four subgroups further weakens the observed outcomes in this study due to the more limited participant numbers in each subgroup. Additionally, because subgroups were determined based on ethnic identity and internalized racism scores, participants who did not respond to both measures were excluded from this subgroup analysis, thus limiting participant numbers further. A larger number of participants in each subgroup could strengthen future studies and lead to more robust observations of differences in identity-related coping behaviors and mental health outcomes between the four groups.

#### Quantitative Limitations

Our utilization of hierarchical regression analyses to detect the impact of potential moderators is accompanied by several limitations. First, the independent variable “racism,” assessing whether a person experienced anti-Asian discrimination after the start of COVID-19, was measured by a yes/no question. Both the binary nature of this variable and the demand characteristics of this measurement further weaken these findings; a continuous, more subtle measure of experienced racism instead could further solidify the results of these hierarchical regression analyses. Additionally, the regression analyses detected only linear relationships, limiting our interpretation of these results. It is possible that the relationship between these variables is nonlinear, which needs to be further explored by future studies. The regression could have also captured outliers it did not intend to. However, our assumption checks prior to performing the moderation analyses should have addressed these common concerns.

Furthermore, the unidimensional nature of the MEIM may have led to the insignificant moderating effects of ethnic identity in this study. Instead, if a multidimensional measurement of ethnic identity were utilized, we could better identify which particular subcomponent best protects against trauma outcomes. For example, the Multidimensional Inventory of Black Identity (MIBI) has been adapted for and validated in Asian American samples [64] and consists of dimensions of ethnic identity such as salience, centrality, ideology, and regard. Future research should consider multidimensional ethnic identity scales such as this one.

Other limitations include the emerging nature of the IRSAA scale for measuring internalized racism, the increased measurement error that accompanies three-way interactions, and potentially missing additional confounding variables that influence the relationship between racism and psychological sequelae. A future study that conducts similar research with the same moderators could be beneficial to support our findings, and additional research looking at other potential risk or protective factors could allow for better understanding of underlying influences affecting Asian American mental health.

## Conclusion

Overall, we found a wide range of nuanced experiences for Asian American individuals during the COVID-19 pandemic. Our participants’ experiences ranged from adaptive to adverse impacts that presented various ethnic identity-promoting and identity-avoidant outcomes and behaviors and varying levels of racial trauma and PTSD following anti-Asian racism. Based on our findings, we conclude that ethnic identity and internalized racism do not seem to serve as moderators, but they may individually influence mental health outcomes and identity-related coping responses. These results can offer providers of Asian American clients a better understanding of identity-related influences on mental health and can better optimize diagnoses and services that are most beneficial for clients. Moreover, findings imply that ethnic identity-promoting initiatives can foster improved engagements at the community level for Asian individuals.

## Data Availability

De-identified data and data analyses will be made available upon reasonable request to the corresponding author, EN.
